# Erk3 deletion drives oxidative adaptations in skeletal muscle

**DOI:** 10.1016/j.molmet.2026.102338

**Published:** 2026-02-24

**Authors:** Angel Loza-Valdes, Carlos Acosta-Gallo, Toufic Kassouf, Andrei Belykh, Małgorzata Stelmach, Dominika Malińska, Katia El Ghoz, Rabih El-Merahbi, Filip Dziaczkowski, Katarzyna Kolczyńska-Matysiak, Grzegorz Sumara

**Affiliations:** 1 Nencki Institute of Experimental Biology, Polish Academy of Sciences, 3 Pasteur Street, 02-093 Warszawa, Poland; 2 Rudolf-Virchow-Zentrum. Center for Integrative and Translational Bioimaging, University of Würzburg, 97080 Würzburg, Germany

**Keywords:** Skeletal muscle, Erk3, Mk5, Oxidative metabolism, Fiber types, Obesity

## Abstract

**Background:**

Skeletal muscle plays a central role in whole-body energy expenditure and metabolic homeostasis, and improving its mitochondrial function and oxidative fiber profile is considered an effective strategy to counteract diet-induced metabolic impairments, although the molecular regulators of these adaptations are not yet fully understood. Erk3 has been implicated in myotube differentiation and in skeletal muscle adaptations to aerobic exercise; however, its potential role in skeletal muscle during diet-induced metabolic dysfunction remains to be determined.

**Methods:**

In this study, we used mice with striated muscle-specific Erk3 deletion alongside *in vitro* cultured myotubes, integrating metabolic phenotyping, indirect calorimetry, multi-omics profiling, and analyses of muscle morphology and fiber-type composition.

**Results:**

Deletion of Erk3 in striated muscle protected mice from diet-induced obesity, glucose intolerance, and insulin resistance, accompanied by increased energy expenditure and elevated mitochondrial content. In cultured myotubes, silencing Erk3 or its putative interaction partner Mapkapk5 (Mk5) enhanced mitochondrial respiration and mitochondrial abundance, particularly under lipid overload. Global transcriptomic and proteomic analyses in myotubes deficient for either Erk3 or Mk5 revealed largely distinct molecular signatures for both kinases. However, consistent with increased oxidative respiration in the absence of Erk3 or Mk5, markers of oxidative fiber types were elevated while glycolic-fiber-specific proteins were diminished in the absence of one or the other kinase. Consistent with these findings, high-fat diet-fed Erk3-deficient mice showed fewer centrally located nuclei and were protected from the fiber-type remodeling associated with metabolic dysfunction.

**Conclusions:**

Our study demonstrates that Erk3 is a key regulator of skeletal muscle oxidative remodeling and metabolic resilience. The deletion of Erk3 in muscles promotes energy expenditure in the myotubes by enhancing mitochondrial function and shifting fiber identity toward oxidative types. Thus, deletion of this kinase protects against high-fat diet–induced obesity, glucose intolerance, and insulin resistance.

## Highlights

• Muscle-specific Erk3 deletion enhances energy expenditure and protects from diet-induced metabolic dysfunction.
• Erk3 deletion increases mitochondrial function and oxidative fiber identity.
• Erk3 and Mapkapk5 shape muscle metabolic programs through distinct pathways.

## Graphical abstract

**Figure:**
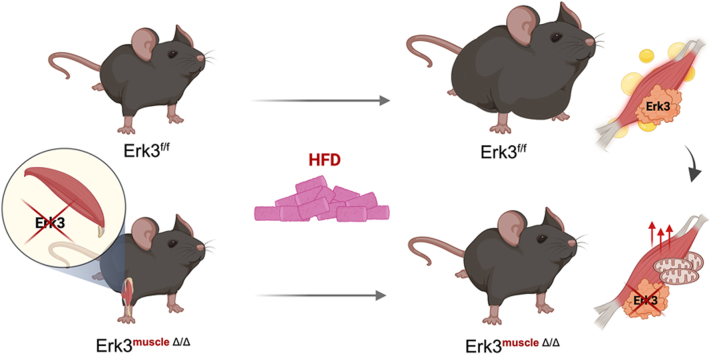

## 1 Introduction

Skeletal muscle plays a central role in systemic energy homeostasis. It accounts for ∼40% of body mass in lean individuals and is a major site of resting and exercise-induced energy expenditure [1,2]. Muscle contraction during physical activity markedly increases energy utilization and, together with dietary restriction, represents one of the most effective interventions to prevent obesity and related metabolic diseases [3]. Skeletal muscle derives energy primarily from glycolysis and oxidative metabolism of glucose and fatty acids in mitochondria. The balance between these pathways is shaped by the muscle's contractile activity pattern, its energetic demands, and the underlying distribution of fiber types [4]. Slow-twitch fibers (type I) are mitochondria-rich, fatigue-resistant, and rely primarily on oxidative metabolism of fatty acids, whereas fast-twitch fibers (type II) depend more heavily on glycolysis. Type II fibers include the oxidative–glycolytic IIA subtype and the predominantly glycolytic IIX subtype. Together with intermediate hybrid fibers such as I–IIA and IIA–IIX, these fiber populations form a continuous phenotypic spectrum that underlies the metabolic flexibility of skeletal muscle [5]. In mice, an additional subtype, type IIB, exhibits extremely rapid contraction and almost exclusive reliance on glycolysis; this fiber type is absent in humans. Importantly, most muscles contain a mixture of fiber types, and their composition can shift in response to environmental, physiological, or pathological cues, including exercise, obesity, and insulin resistance [6,7]. Such remodeling affects systemic metabolism and insulin sensitivity, whereas inactivity, obesity, and diabetes promote inflammation, impaired mitochondrial biogenesis and function, and a shift from oxidative to glycolytic fibers. These processes contribute to muscle weakness (dynapenia) may accelerate the development of sarcopenia [6,[8], [9], [10], [11]]. Despite the importance of muscle metabolism for whole-body energy balance, no pharmacological therapies are currently available which would both ameliorate obesity and improve skeletal muscle function.

Extracellular signal-regulated kinase 3 (Erk3, also known as MAPK6) is an atypical member of the MAPK family that undergoes rapid proteasomal degradation unless stabilized by complex formation with MAP kinase-activated protein kinase 5 (Mapkap5, referred here as Mk5) [12]. We recently showed that stabilization of the Erk3-Mk5 complex in adipocytes promotes FOXO1-dependent transcription of adipose triglyceride lipase (ATGL) in response to adrenergic stimulation, thereby enhancing lipolysis. Conversely, Erk3 deletion in adipocytes increased oxygen consumption in white/beige and brown adipocytes, suggesting elevated mitochondrial activity [13]. Erk3 levels increase substantially during the differentiation of C2C12 myocytes into myotubes [14]. Moreover, aerobic exercise training has been shown to reduce its expression in skeletal muscle [15], suggesting that Erk3 may contribute to skeletal muscle adaptation to increased aerobic metabolic demand. Whereas the kinase activity of Mk5 displays sex-dependent regulation in human skeletal muscle, which might lead to differential insulin sensitivity in females and males [16]. In line, recent evidence suggests that blood circulating levels of Mk5, correlate with glucose levels in diabetic patients [17]. Given the central role of skeletal muscle in whole-body energy expenditure, we investigated whether striated muscle-specific Erk3 deletion alters susceptibility to diet-induced obesity and metabolic disturbances. We demonstrate that Erk3 ablation in striated muscle protects mice from high-fat diet (HFD)-induced obesity, glucose intolerance, and insulin resistance, and these effects are associated with increased energy expenditure, enhanced mitochondrial content, and a shift toward oxidative muscle fibers. These findings suggest that targeting Erk3 in skeletal muscle may represent a promising strategy for the development of future therapies against obesity and related metabolic disorders.

## 2 Materials and methods

### 2.1 Mice

For targeted constitutive deletion of Erk3 in striated muscles (Erk3^muscleΔ/Δ^), Erk3^f/f^ [13] mice were crossed with C57BL/6J mice expressing Cre recombinase under the muscle creatine kinase (MCK) promoter [18]. Experiments were performed exclusively in male mice to minimize variability associated with hormonal fluctuations during the estrous cycle. Erk3^f/f^ littermates were used as controls. Mice were held under specific pathogen-free conditions in the animal facility of Nencki Institute of Experimental Biology on a 12:12 light: dark cycle and allowed free access to a standard chow diet or HFD (ResearchDiets, D12331i) and water. HFD was initiated immediately after weaning, at 4 weeks of age. Bodyweight gain of mice was recorded weekly. At the end of the experiment, terminal body composition was assessed by NMR. Subsequently, mice were euthanized for organ dissection. Mouse experiments were approved by the local ethics committee of Warsaw (I Lokalna Komisja Etyczna ds. Doświadczeń na Zwierzętach, reference: 976/2020).

### 2.2 *In vivo* metabolic tests

For glucose tolerance tests, mice were fasted overnight before receiving an intraperitoneal injection of glucose (2 g/kg) after 16 weeks in HFD. Insulin tolerance tests were performed after a 4-hour fast using intraperitoneal administration of human insulin (0.5 U/kg) after 17 weeks in HFD. Blood glucose levels were measured from tail-tip samples using an Accu-Chek glucometer (Roche) immediately prior to injection and at 15, 30, 60, 90, and 120 min thereafter.

### 2.3 Metabolic phenotyping

Indirect calorimetry was performed using a Phenomaster system (TSE Systems). Mice were housed individually and maintained on a controlled 12 h light/12 h dark cycle at an ambient temperature of 22 °C, with ad libitum access to food and water. Prior to data collection, animals were acclimatized to single housing conditions and drinking bottles for at least 48 h. Food and water intake, locomotor activity (assessed by photobeam breaks), oxygen consumption (VO_2_), carbon dioxide production (VCO_2_), energy expenditure (EE), and respiratory exchange rate (RER) were continuously recorded at 10-minute intervals. Measurements were conducted over a period of at least six full, consecutive days. EE and RER were calculated from VO_2_ and VCO_2_ values obtained by indirect calorimetry as described previously [19]. Analyses were performed separately for data collected during the light phase (day), dark phase (night), and during resting conditions. Resting state was defined as 10-minute intervals with locomotor activity below 5 activity counts. For each mouse and condition, the collected measurements of EE, RER, food intake, and locomotor activity were summarized into a single representative value, and each animal was treated as one independent observation.

### 2.4 Cell culture

C2C12 myoblasts were maintained in growth medium (DMEM high glucose) supplemented with 10% fetal bovine serum, 1% penicillin–streptomycin, and 1% L-glutamine at 37 °C and 5% CO_2_. Medium was replaced every two days, and cultures were kept below 70% confluency to prevent premature fusion and preserve the myoblast population. Myogenic differentiation was induced by switching to differentiation medium (DMEM high glucose supplemented with 2% horse serum, 1% L-glutamine, and 1% penicillin–streptomycin). Cells were used for downstream experiments after six days of differentiation, unless stated otherwise. For experiments employing palmitic acid treatment, differentiated myotubes were incubated for 16 h in serum-free medium containing 0.5 mM palmitic acid conjugated to BSA, while control cells were treated with the corresponding BSA vehicle.

### 2.5 Transient transfection with siRNA

Undifferentiated C2C12 myoblasts at full confluence were transfected with siRNAs using DharmaFECT Duo (Dharmacon). siRNAs and transfection reagent were diluted separately in Opti-MEM, mixed, and incubated for 30 min at room temperature before being added directly to the cells in differentiation medium. Reagent amounts were normalized to cell growth area, corresponding to 2.1 μL/cm^2^ DharmaFECT Duo and 1.25 nM/cm^2^ siRNA. All siRNAs were used as SMARTpool reagents (Dharmacon).

### 2.6 Adenovirus infection

Adenoviral infection of C2C12 cells was performed 4 days after initiating differentiation. Cells were transduced with adenoviruses expressing enhanced green fluorescent protein or wild-type Erk3 at a multiplicity of infection of 400. Medium was replaced the following day, and cells were used for experiments 48 h after infection.

### 2.7 Oxygen consumption

Mitochondrial respiration in differentiated C2C12 myotubes was evaluated using the Seahorse XFe96 Analyzer and the XF Cell Mito Stress Test kit (Agilent, 103015-100), following the manufacturer's guidelines. Briefly, before the measurement, cells were incubated for 1 h in Seahorse assay medium. The assay consisted of sequential injections of 2 μM oligomycin, 1 μM FCCP, and 0.75 μM rotenone/antimycin A to probe distinct components of the mitochondrial respiratory chain. Basal respiration was calculated as the oxygen consumption rate (OCR) measured prior to oligomycin injection, corrected for non-mitochondrial respiration determined after rotenone/antimycin A. ATP-linked respiration was calculated as the difference between basal OCR and OCR following oligomycin injection. Maximal respiration was defined as the peak OCR after FCCP injection, corrected for non-mitochondrial respiration, and spare respiratory capacity was calculated as the difference between maximal and basal respiration. After the run, cells were fixed, and total DNA content was quantified using crystal violet staining and used to normalize OCR values across wells.

### 2.8 MitoTracker staining

C2C12 myotubes grown on coverslips were incubated with 100 nM MitoTracker Red dye (Thermo Fisher) diluted in pre-warmed culture medium for 30 min at 37 °C, protected from light. After staining, cells were washed with PBS and fixed with 3.7% paraformaldehyde for 15 min. Nuclei were counterstained with DAPI before mounting with ProLong Gold Antifade. Images were acquired with Microscopes: Upright Olympus VS110; Camera Hamamatsu ORCA Flash4.0 V2 2048 × 2048 pixels, software OLYMPUS VS-ASW and Inverted Leica DMI8, CAMERA LEICA DFC365 FX. Images were analyzed with ImageJ/Fiji.

### 2.9 C2C12 immunostaining

C2C12 myotubes grown on coverslips were fixed with 3.7% paraformaldehyde for 15 min, permeabilized with 1% Triton X-100 in PBS for 10 min, and blocked with 1% goat serum in 0.05% PBS-Tween for 30 min. Cells were incubated overnight at 4 °C with anti-ATP5A1 primary antibody (Thermo Fisher, 459000) diluted in blocking solution, followed by 1 h incubation at room temperature with fluorophore-conjugated donkey secondary antibodies. Nuclei were counterstained with DAPI before mounting with ProLong Gold Antifade. Images were acquired with Microscope: Upright Olympus VS110; Camera Hamamatsu ORCA Flash4.0 V2 2048 × 2048 pixels, software OLYMPUS VS-ASW and Inverted Leica DMI8, CAMERA LEICA DFC365 FX. Images were analyzed with ImageJ/Fiji.

### 2.10 Histological, geometrical, and fiber-type distribution analyses

Tissues were embedded in OCT and rapidly frozen in isopentane cooled with liquid nitrogen. For morphological assessment, 10-μm cryosections were stained with hematoxylin-eosin (H&E) according to the standard procedure. Fiber type distribution was analyzed using the method adapted from Wojton et al. [20]. Briefly, cryosections were incubated overnight at 4 °C with a primary antibody cocktail recognizing MyHC I (DSHB, BA-F8), MyHC IIa (DSHB, SC-71), MyHC IIb (DSHB, BF–F3), and laminin (Merck, L8271). After washing, sections were incubated with a secondary antibody mixture consisting of Alexa Fluor 488 goat anti-mouse IgG1, Alexa Fluor 555 goat anti-mouse IgM, and Alexa Fluor 633 goat anti-mouse IgG2b (Thermo Scientific). Laminin staining was used to quantify muscle fiber geometry, including cross-sectional area, Feret diameter, and minimum Feret diameter. Images were acquired using an Olympus VS110 fluorescent slide scanner equipped with an RGB camera and a 20 × /0.75 NA objective. Fiber-type distribution and fiber geometry were quantified using ImageJ/Fiji.

### 2.11 Western blotting

Total protein was extracted from cells and tissues using RIPA buffer supplemented with protease and phosphatase inhibitor cocktails. Tissues were homogenized with a tissue lyser, whereas cultured cells were scraped and passed through a 26-gauge needle to ensure complete lysis. Lysates were cleared by centrifugation (13,000 rpm, 10 min, 4 °C), and supernatants were collected. Protein concentration was determined using a BCA assay. Equal amounts of protein were mixed with loading dye, heated at 95 °C for 5 min, resolved on 10% SDS–PAGE gels, and transferred onto PVDF membranes (Millipore). Membranes were blocked in 5% skim milk in TBS buffer with 0.1% Tween 20, incubated overnight at 4 °C with primary antibodies, washed, and incubated with HRP-conjugated secondary antibodies. For immunoblotting analyses, the following primary antibodies were used: α-ERK3 (Abcam, ab53277-100), α-MK5 (Abcam, ab93800), α-ATP5A (Abcam, 14748), α-OXPHOS (Abcam, 10413 [MS604-300]), α-FOXO1 (Cell Signaling, 2880S), α-p-FOXO1 (Thr24) (Cell Signaling, 94645), α-FOXO3a (Cell Signaling, 2497s), α-p-FOXO3a (Ser413) (Cell Signaling, 8174S), α-GAPDH (Sigma, G9545), α-MYHC (Bio-Techne, MAB4470), α-desmin (Cell Signaling, 5332T), α-vinculin (Cell Signaling, 13901S), α-MYH4 (DSHB, BF–F3), α-MYH7 (DSHB, BA-D5), α-MYH2, α-(DSHB, SC-71), α-MYH1 (DSHB, 6H1).

### 2.12 RNA-seq analysis

Total RNA was isolated from C2C12 cells using TRIzol (Invitrogen) following the manufacturer's instructions. RNA quality and integrity were evaluated on an Agilent 2100 Bioanalyzer with the RNA 6000 Nano Kit, and only high-quality samples were taken for further analysis. Strand-specific, poly(A)-selected libraries were generated from 500 ng of total RNA using the KAPA Stranded mRNA Sample Preparation Kit. Briefly, mRNA was captured with poly-T–coated magnetic beads, fragmented, and converted into first-strand cDNA. Second-strand synthesis produced double-stranded cDNA, which was subsequently A-tailed and ligated to sequencing adapters (NEB). Adapter uracils were removed with USER enzyme prior to PCR amplification. Library size distribution (∼300 bp) was assessed using the Agilent High Sensitivity DNA kit, and concentrations were measured using the QuantiFluor dsDNA system (Promega). Libraries were sequenced on an Illumina NovaSeq 6000 platform in 2 × 151 bp paired-end mode.

Initial read quality was assessed with FastQC (v0.11.9). Adapter sequences and low-quality bases were removed using cutadapt (v3.4) and TrimGalore (v0.6.7; quality cutoff 25). Cleaned reads were aligned to the mouse reference genome (GRCm39) using STAR (v2.7.9a) with default settings and Ensembl release 105 annotation. Duplicate reads were marked using Picard MarkDuplicates (v2.27.4-SNAPSHOT). MultiQC (v1.13) was used to summarize quality metrics across all samples. Gene-level counts were obtained with featureCounts (v2.0.0), retaining only primary, reversely stranded, paired-end alignments with mapping quality ≥3.

All downstream analyses were performed in R (v4.1.3). Differential expression analysis was conducted with DESeq2 (v1.34) using default parameters. Features with fewer than 10 total counts across all samples were removed prior to model fitting. Differentially expressed genes were defined based on a false discovery rate (FDR, padj) below 0.05 and an absolute fold change greater than 1.3. Functional enrichment of significant genes was carried out using g:Profiler with a focus on Gene Ontology Biological Process (GO:BP) terms.

### 2.13 Proteomic analysis

Protein extracts were processed using the SP3 workflow (PMID: 25358341) implemented on the KingFisher Apex™ system (Thermo Fisher). Proteins were digested with trypsin at a 1:20 enzyme-to-substrate ratio in 50 mM HEPES containing 5 mM TCEP and 20 mM CAA, and the reaction proceeded for 5 h at 37 °C. Up to 10 μg of the resulting peptides were labeled with TMT10plex™ reagents following a previously established protocol (PMID: 24579773). In brief, 0.8 mg of TMT reagent was dissolved in 45 μL of anhydrous acetonitrile, and 4 μL of this solution was added to each sample. After 1 h of incubation at room temperature, the reaction was quenched with 4 μL of 5% hydroxylamine, followed by a 15 min incubation. Labeled peptides were pooled, desalted using an Oasis® HLB μElution Plate (Waters), and concentrated by vacuum centrifugation.

Peptide separation was carried out using the following gradient: 2–8% B in 4 min, 8–28% B over 104 min, 28–40% B in 4 min, 40–80% B in 4 min, and re-equilibration at 2% B for 4 min. Eluted peptides were introduced into an Orbitrap Fusion™ Lumos™ Tribrid™ mass spectrometer (Thermo Fisher Scientific) via a Pico-Tip emitter (360 μm OD × 20 μm ID; 10 μm tip, CoAnn Technologies) with a spray voltage of 2.4 kV. The capillary was maintained at 275 °C. Full MS scans were recorded in profile mode over *m*/*z* 375–1500 at 120,000 resolution (*m*/*z* 200). The maximum injection time was 50 ms and the AGC target was set to “standard.” Data-dependent acquisition was employed, and MS/MS spectra were collected in the Orbitrap at 30,000 resolution with a 94 ms maximum injection time and an AGC target of 200%. Fragmentation was achieved via HCD with 36% normalized collision energy. The quadrupole isolation width was 0.7 *m*/*z*, dynamic exclusion was set to 60 s, and precursor charge states 2–7 were selected for fragmentation. Raw MS data were converted to mzML format using ProteoWizard MSConvert with peak picking enabled, 64-bit encoding, zlib compression, and retention of the 1000 most intense peaks. Database searches were carried out in MSFragger through FragPipe (18.0-build13) against the FASTA database UP000000589_MusMusculus_C57BL_ID10090_21968entries_27102022_dl11012023, supplemented with common contaminants and decoy sequences. Carbamidomethylation of cysteine and TMT modification of lysine were set as fixed modifications, whereas methionine oxidation, protein N-terminal acetylation, and TMT at peptide N-termini were included as variable modifications. Mass tolerances were set to 20 ppm for both MS1 and MS2 scans. Trypsin specificity was enforced, allowing up to two missed cleavages, and peptides shorter than seven amino acids were excluded. The false discovery rate was controlled at 1% for both peptides and proteins.

For downstream proteomic analyses, protein.tsv output files from FragPipe were processed in R (ISBN 3-900051-07-0). Contaminants and decoy entries were removed, and only proteins quantified by at least two razor peptides were retained, yielding 1447 high-confidence proteins. To mitigate technical variation, batch effects were corrected on log2-transformed TMT reporter intensities using the ‘removeBatchEffect’ function from the limma package (PMID: 25605792), followed by variance stabilization normalization using ‘normalizeVSN’ (PMID: 12169536). Differential abundance analysis was performed with limma's moderated t-tests, incorporating replicate structure into the design matrix. P-values and FDRs (padj) were obtained using the ‘fdrtool’ package (PMID: 18441000). Proteins were considered significantly altered if they exhibited an FDR <0.05 and an absolute fold change >1.3.

### 2.14 Statistical analysis of non-omics experiments

Data are presented as mean ± standard error of the mean (SEM). Statistical significance was assessed using two-tailed Student's t-tests for comparisons between two groups, or one-way/two-way analysis of variance (ANOVA) followed by Tukey's post hoc test for multiple comparisons. A p-value of <0.05 was considered statistically significant (∗p < 0.05, ∗∗p < 0.01, ∗∗∗p < 0.001).

## 3 Results

### 3.1 Striated muscle-specific Erk3 ablation protects against diet-induced obesity and glucose intolerance

**Figure 1:**
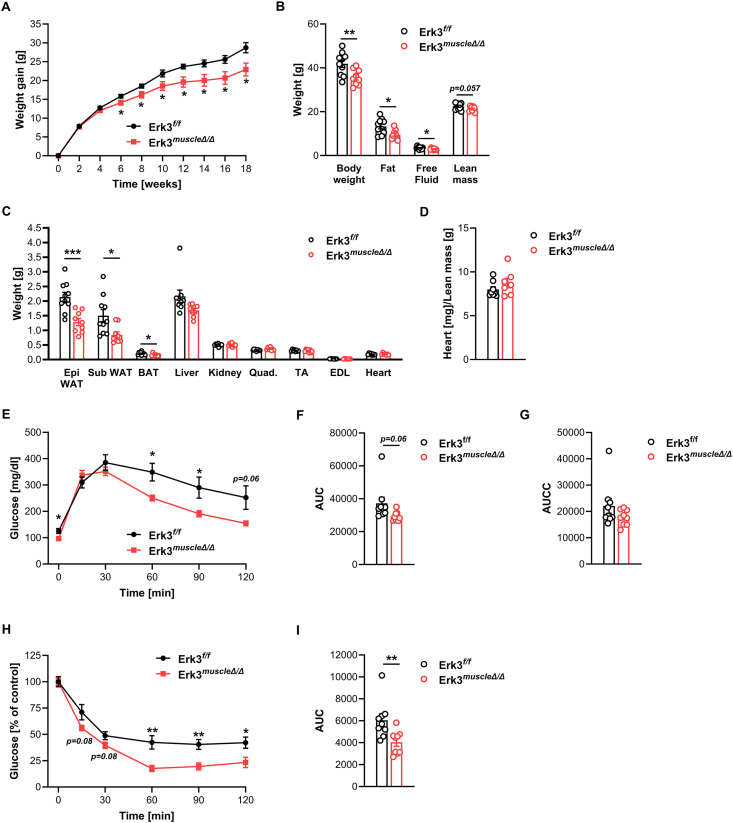
**Striated muscle-specific Erk3 ablation protects against diet-induced obesity and glucose intolerance.** Body weight gain (A), body composition (B), organ and tissue weight (C,D), glucose tolerance test (E–G), and insulin tolerance test (H,I) in Erk3^muscleΔ/Δ^ mice and control littermates after 18 weeks of HFD feeding (from 4 to 22 weeks of age). Erk3^muscleΔ/Δ^, n = 10 (A–C), n = 8 (D) or n = 9 (E–I). Erk3^f/f^, n = 9 (A-C, E-I) or n = 7 (D). AUC - total area under the curve, AUCC - incremental area under the curve. Each n represents a sample from distinct mice. Data presented as mean ± SEM. ∗p < 0.05, ∗∗p < 0.01, ∗∗∗p < 0.001. Unpaired two-tailed Student's t-test.

Our previous studies demonstrated that targeted deletion of Erk3 in mouse adipocytes enhances energy expenditure, leading to a lean phenotype and improved glucose homeostasis [13]. Since skeletal muscles are major contributors to whole-body energy expenditure, we investigated whether striated muscle-specific deletion of Erk3 affects diet-induced weight gain and systemic metabolism. To this end, we utilized mice with Erk3 constitutive knockout driven by the MCK-Cre promoter (Erk3^muscleΔ/Δ^), resulting in ablation of this kinase in both skeletal muscle and heart tissue (Fig. S1A–D). Mice were fed a high-fat diet (HFD) for 18 weeks. Starting from week 8 of HFD feeding, Erk3^muscleΔ/Δ^ mice exhibited significantly reduced body weight gain compared to control littermates (Fig. 1A). This reduction in body mass was mainly attributable to decreased fat mass and a near-significant trend toward lower lean mass (Fig. 1B). Erk3^muscleΔ/Δ^ mice displayed markedly lower weights of white adipose tissue (subcutaneous and epididymal) as well as brown adipose tissue, while the weights of the quadriceps, tibialis anterior (TA), extensor digitorum longus (EDL), and heart were unchanged (Fig. 1C–D). As expected, HFD feeding in control mice led to increased adiposity accompanied by impaired glucose utilization and insulin resistance. Strikingly, Erk3^muscleΔ/Δ^ mice were protected from these metabolic disturbances: glucose tolerance tests and insulin tolerance tests revealed significantly improved glucose tolerance and insulin sensitivity in parallel with reduced adiposity (Fig. 1E–I). Notably, Erk3^muscleΔ/Δ^ mice maintained on a standard chow diet showed no significant alterations in body composition, organ weights, or glucose tolerance in either adult or aged cohorts (Fig. S1E-N). Together, these results demonstrate that striated muscle-specific Erk3 ablation protects against diet-induced obesity and associated defects in glucose homeostasis.

### 3.2 Erk3 ablation in skeletal muscle leads to increased energy expenditure and enhanced mitochondrial content

**Figure 2:**
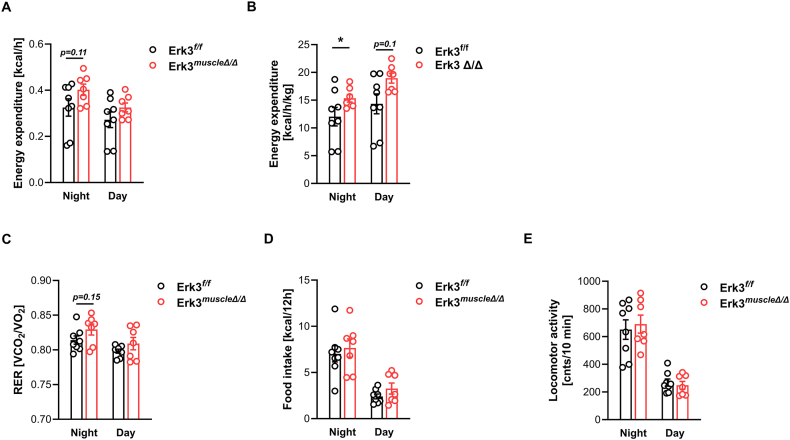
**Erk3 ablation in skeletal muscle increases energy expenditure in mice**. Unnormalized (A) and lean mass-normalized (B) energy expenditure, respiratory exchange ratio (C), food intake (D), and voluntary locomotor activity (E) in Erk3^muscleΔ/Δ^ mice (n = 7) and control littermates Erk3^f/f^ (n = 8) after 18 weeks of HFD feeding (from 4 to 22 weeks of age). RER - respiratory exchange ratio, cnts - counts. Each n represents a sample from distinct mice. Data presented as mean ± SEM. ∗p < 0.05. Unpaired two-tailed Student's t-test.

Increased adiposity results from a sustained positive energy balance, where caloric intake chronically exceeds energy expenditure. To determine whether Erk3 ablation in striated muscle influences whole-body energy expenditure, we performed indirect calorimetry in HFD-fed Erk3^muscleΔ/Δ^ mice. Unadjusted energy expenditure showed a strong trend toward higher values in HFD-fed Erk3^muscleΔ/Δ^ mice during night, but not day. Interestingly the trend towards elevated energy expenditure was also observed during the resting state (Fig. 2A, S2A-B). In relation to lean mass, mice deficient for Erk3 in muscle presented significantly elevated energy expenditure during the dark phase, and a trend during the day and resting stage (Fig. 2B, S2C-D). The trend towards increased energy dissipation by Erk3^muscleΔ/Δ^ mice was associated with the tendency for increased utilization of carbohydrates as a fuel, as indicated by increased respiratory exchange ratio primarily during night but not during the day and the resting state (Fig. 2C, S2E-F). However, food intake and locomotor activity was not affected by Erk3 deletion (Fig. 2D–E), suggesting an intrinsic alteration in skeletal muscle energy metabolism.

**Figure 3:**
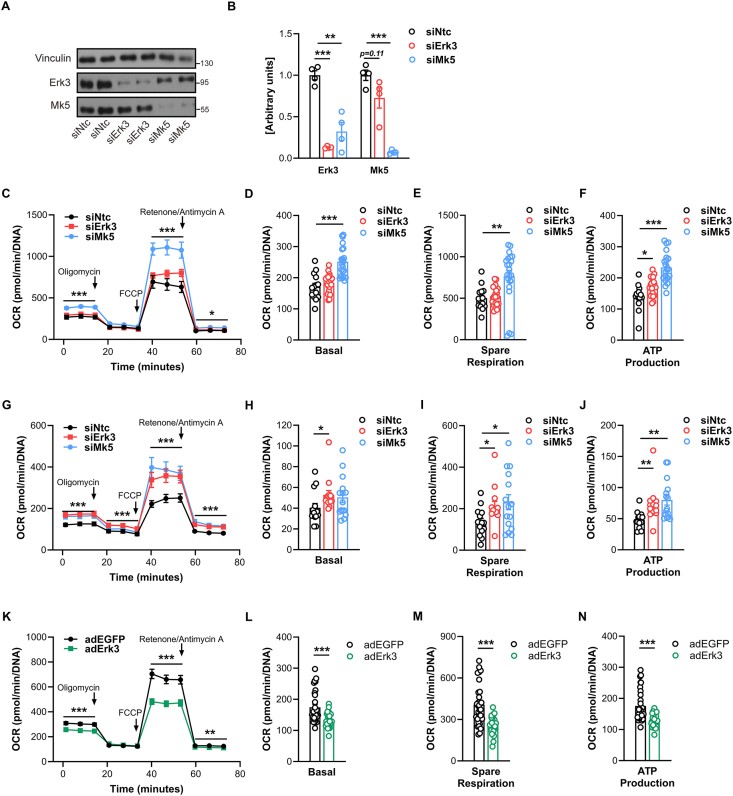
**Erk3 and Mk silencing alters mitochondrial respiration in C2C12 cells.** Representative immunoblots (A) with quantification (B) of indicated proteins in differentiated C2C12 cells after silencing of Erk3 or Mk5 genes. Seahorse analysis of mitochondrial respiration rate (C,G,K), basal (D,H,L) and spare respiration level (E,I,M), ATP production (F,J,N) in C2C12 cells following Erk3 (siErk3), Mk5 (siMk5) silencing under basal condition (C–F) or after 16h treatment of 0.5 mM palmitic acid (G–J), or following overexpression of Erk3 (adErk3) or EGFP (adEGFP) under basal condition (K–N). siNtc (non-targeting control), n = 4 (A–B), n = 14 (C–F), n = 16 (G–J). siErk3, n = 4 (A–B), n = 21 (C–F), n = 14 (G–J). siMk5, n = 4 (A–B), n = 21 (C–F), n = 15 (G–J). adEGFP, n = 30 (K–N). adErk3, n = 27 (K–N). Data presented as mean ± SEM. ∗p < 0.05, ∗∗p < 0.01, ∗∗∗p < 0.001. Unpaired two-tailed Student's t-test.

We previously reported that Erk3 forms a complex with Mk5 in adipocytes, which stabilizes both kinases and prevents their degradation [13]. To further dissect this pathway, we analyzed the effects of Erk3 or Mk5 silencing in C2C12 myotubes. Silencing of Mk5 reduced Erk3 protein levels, whereas silencing of Erk3 resulted in a non-significant trend toward reduced Mk5 protein abundance in C2C12 cells (Fig. 3A–B). To investigate whether changes in mitochondrial function might underlie the observed phenotype in Erk3^muscleΔ/Δ^ mice, we performed Seahorse analysis of mitochondrial respiration rate. Interestingly, silencing of Erk3 alone did not alter the oxygen consumption rate (OCR), while Mk5 silencing significantly increased basal respiration, spare respiratory capacity, and ATP production-related respiration (Fig. 3C–F). However, when cells were additionally challenged with palmitic acid to mimic lipid overload, silencing of Erk3, similarly to silencing of Mk5, increased basal OCR, spare respiration, and ATP production (Fig. 3G–J). These findings indicate that under lipotoxic stress, disruption of the Erk3-Mk5 axis enhances mitochondrial activity, which may explain the phenotype observed *in vivo* under HFD conditions. Moreover, they suggest that Mk5 may regulate skeletal muscle metabolism independently of Erk3. Consistent with this, overexpression of Erk3 in C2C12 myotubes reduced basal OCR, spare respiratory capacity, and ATP production compared to control cells (Fig. 3K-N), supporting the concept that Erk3 regulates mitochondrial activity.

**Figure 4:**
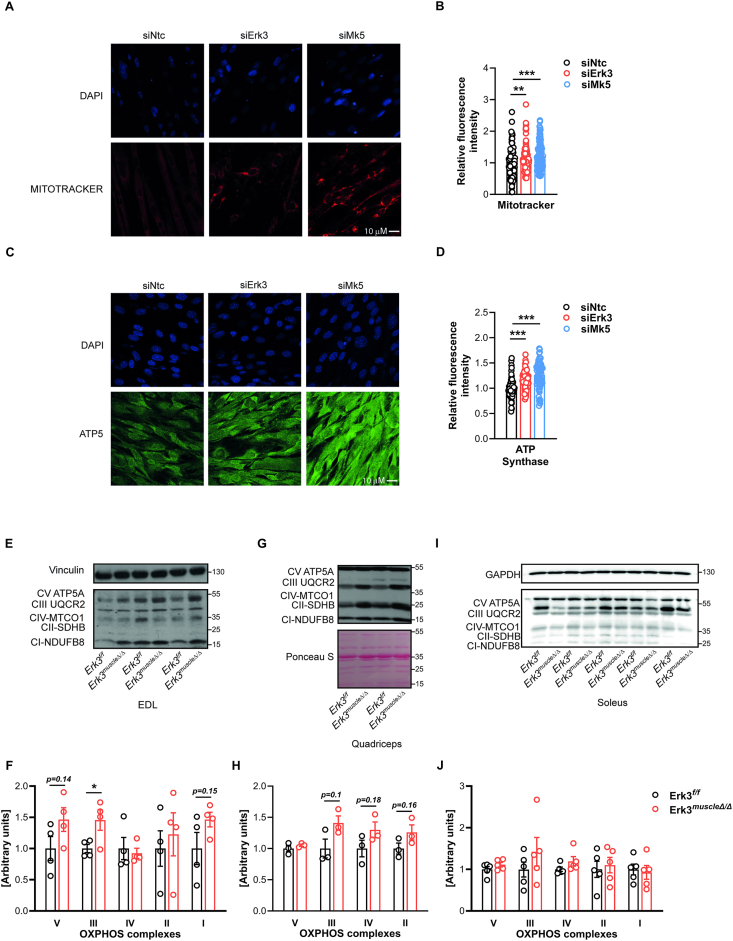
**Erk3 ablation enhances mitochondrial content in skeletal muscle**. Representative microscopy pictures (A,C) and quantification of relative fluorescence intensity (B,D) of C2C12 myotubes after Erk3 (siErk3) or Mk5 (siMk5) silencing, followed by MitoTracker Red staining (A,B) or ATP synthase (C,D). Representative immunoblots (E,G,I) and quantification (F,H,J) of OXPHOS complex protein content in extensor digitorum longus muscle (EDL) (E–F), quadriceps (G–H), and soleus (I–J) muscle from Erk3^muscleΔ/Δ^ mice and control littermates Erk3^f/f^ after 18 weeks of HFD feeding (from 4 to 22 weeks of age). siNtc (non-targeting control), n = 137 (A–B), n = 66 (C–D). siErk3, n = 137 (A–B), n = 65 (C–D). siMk5, n = 106 (A–B), n = 70 (C–D). EDL, n = 4. Quadriceps, n = 3. Soleus, n = 5. Each n represents randomly selected image (A–D) or different animal (E–J). Data presented as mean ± SEM. ∗p < 0.05, ∗∗p < 0.01, ∗∗∗p < 0.001. Unpaired two-tailed Student's t-test.

To further characterize the increase in mitochondrial function, we performed MitoTracker and ATP5 staining to quantify mitochondrial abundance in C2C12 cells. Silencing of either Erk3 or Mk5 increased mitochondrial membrane potential (Fig. 4A,B) and mitochondrial content (Fig. 4C,D). In line with these findings, mitochondrial OXPHOS complex I–V were elevated in EDL, a glycolytic, fast-twitch muscle from Erk3^muscleΔ/Δ^ mice compared to controls. This trend reach significance for the marker of complex III in EDL of the mice deficient for Erk3 (Fig. 4E,F). In contrast, the quadriceps muscle, which contains mixed fiber types, displayed a similar upward trend that did not reach statistical significance (Fig. 4G,H), while no changes were observed in the oxidative soleus muscle (Fig. 4I,J). These results indicate that Erk3 ablation preferentially affects mitochondrial protein content in fast-twitch skeletal muscle.

### 3.3 Erk3 ablation in skeletal muscle promotes a shift toward oxidative muscle fibers

**Figure 5:**
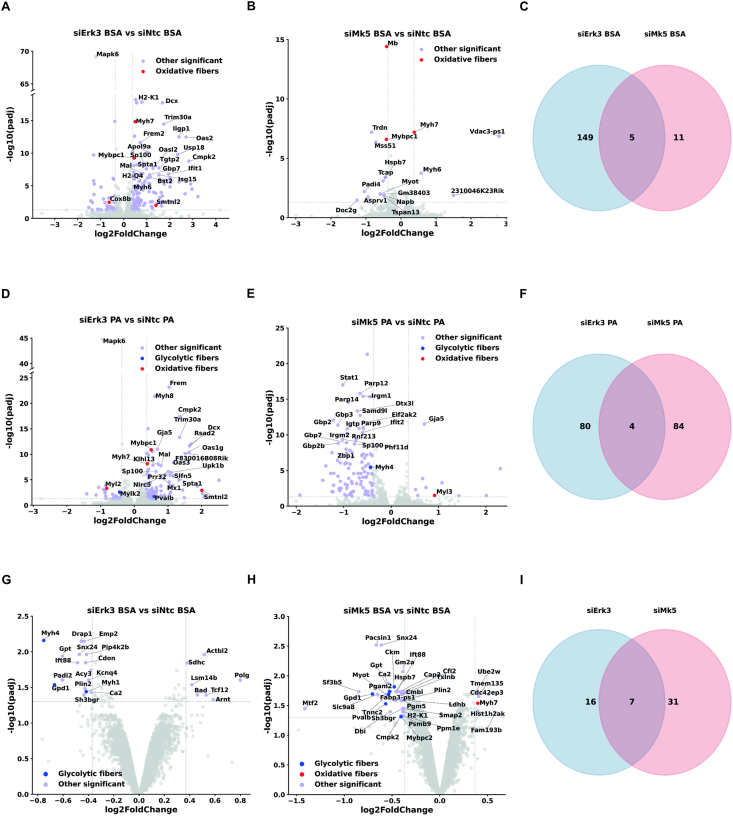
**Erk3 and Mk5 silencing alters gene and protein expression in C2C12 myotubes.** Transcriptomic (A–F) and proteomic (G–I) analyses of C2C12 cells after Erk3 (siErk3) (A, C, D, G) or Mk5 (siMk5) (B, C, E, H) silencing under BSA (A–C, G–I) or 0.5 mM palmitic acid (PA) (D–F) treatment for 16 h, presented as volcano plots (A, B, D, E, G, H) and Venn diagrams (C, F, I) illustrating shared and distinct sets of up- and down-regulated genes/proteins regulated by Erk3 and Mk5, with directionally concordant functional shifts. For transcriptomic datasets, only genes characteristic of oxidative or glycolytic fiber types, along with 20 additional genes with the highest -log10 (padj) value, are labeled. For proteomic datasets, all proteins meeting significance criteria (padj< 0.05 and |log_2_ fold change| ≥ 0.37) are annotated.

In our previous work, we showed that in adipocytes, the Erk3-Mk5 pathway promotes nuclear translocation of FOXO1, a transcription factor that plays a central role in energy homeostasis, including the regulation of insulin signaling and glucose metabolism [13]. However, in skeletal muscle of HFD-fed Erk3^muscleΔ/Δ^ mice, total FOXO1 protein levels, as well as levels of its phosphorylated form (Thr24), were unchanged (Fig. S3A and B). Alongside FOXO1, FOXO3 is highly expressed in skeletal muscle, where it regulates autophagy and mitophagy. Similarly, both total FOXO3 protein levels and its phosphorylated form (Ser413) remained unaltered in HFD-fed Erk3^muscleΔ/Δ^ mice (Fig. S3A and B). Together, these results suggest that Erk3 does not modulate skeletal muscle metabolism through either FOXO1 or FOXO3. Thus, to investigate the molecular basis underlying the protective effect of striated muscle-specific Erk3 ablation, we performed transcriptomic and proteomic profiling of C2C12 myotubes under palmitic acid challenge or control conditions (bovine serum albumin, BSA) following Erk3 or Mk5 silencing. In the RNA-seq dataset, Erk3 expression was significantly reduced upon siErk3 treatment under both conditions, confirming effective silencing at the transcription level (Fig. 5A,C). Mk5 silencing produced a consistent downward trend in Mk5 mRNA levels, although this decrease did not meet the predefined FDR threshold. In the proteomic dataset, neither ERK3 nor MK5 was detected. Efficient silencing of both targets for these experiments was independently validated by Western blot analysis, confirming that the transcriptional and proteomic analyses were performed after verified Erk3/Mk5 silencing (Fig. 3A,B). Importantly, the levels of differentiation markers did not differ significantly following gene silencing, indicating that cells from all groups were differentiated to a comparable extent prior to sample collection (Fig. S3C–D).

To gain insight into the biological programs affected by Erk3 and Mk5 loss, we conducted Gene Ontology Biological Process (GO:BP) enrichment analysis using g:Profiler. GO enrichment of transcriptomic datasets indicated that silencing of Erk3 and Mk5 influenced processes related to the contractile apparatus, including myosin filament organization, sarcomere assembly, and regulation of muscle contraction (Fig. S4A). Beyond muscle-specific pathways, Erk3 depletion also impacted broader regulatory programs, including mainly processes linked to immune or stress responses. By comparison, Mk5 depletion predominantly enriched immune-related pathways, including antiviral defense responses, reactions to external biotic stimuli, and phosphatidylinositol-bisphosphate metabolic processes. Focusing on pathways characteristic for skeletal muscle cells, Erk3 and Mk5 ablation altered the expression of genes characteristic of distinct muscle fiber types. Under basal conditions, loss of either kinase increased the expression of genes typical of oxidative fibers (Fig. 5A–B). Following palmitic acid treatment, Erk3 silencing similarly upregulated oxidative fiber-associated genes and downregulated glycolytic fiber-associated genes (Fig. 5D). Mk5 depletion elicited comparable responses following palmitic acid challenge, albeit involving fewer genes (Fig. 5E).

Using a single-shot LC–MS/MS workflow with a 120-minute gradient, we quantified 1,447 high-confidence proteins. This approach yielded a smaller but highly reliable dataset, prioritizing confidence and quantitative robustness over maximal protein coverage. Proteomic analysis likewise demonstrated reduced abundance of proteins characteristic of glycolytic fibers in response to Erk3 or Mk5 silencing (Fig. 5G–H). Furthermore, protein levels of perilipin-2 (PLIN2), which is implicated in intramyocellular lipid accumulation [21], were markedly reduced upon Erk3 or Mk5 silencing (Fig. 5G–H). These findings indicate that Erk3 and Mk5 influence components of the contractile machinery. Given the small number of differentially abundant proteins, pathway enrichment analysis was not included to avoid overinterpretation. Importantly, comparative analyses of transcriptomic and proteomic data identified relatively few shared differentially expressed genes and proteins (Fig. 5C,F,I). Consistently, principal component analysis (PCA) of the proteomic datasets revealed a clear separation between siErk3-and siMk5-treated samples (Fig. S4B), indicating that Erk3 and Mk5 may regulate skeletal muscle through largely distinct targets and mechanistically independent pathways.

**Figure 6:**
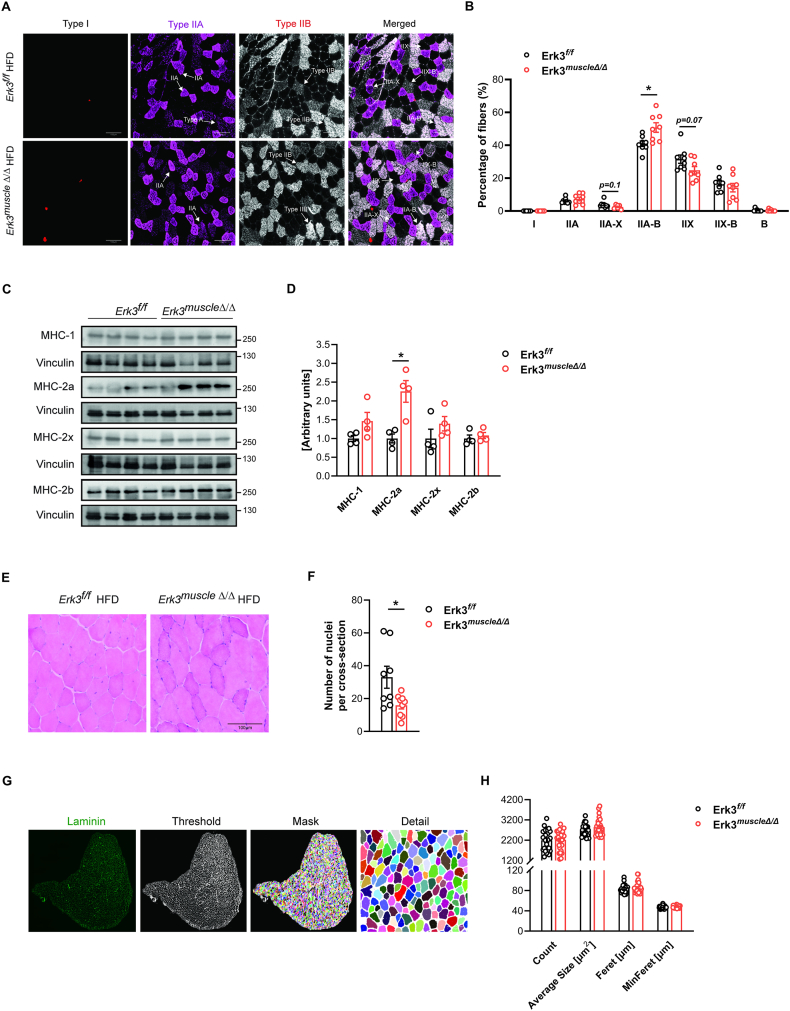
**Erk3 ablation protects against HFD-induced fiber-type switch and muscle remodeling.** Representative pictures (A,C,E,G) with quantification (B,D,F,H) of immunostaining for myosin heavy chain (MHC) isoforms showing fiber-type distribution (A,B), immunoblots of MHC isoforms content (C,D), H&E staining showing centrally located nuclei (E,F), immunostaining of laminin and segmentation workflow (raw signal, thresholded image, fiber mask, and detail view) (G,H), in tibialis anterior muscle of Erk3^muscleΔ/Δ^ and control littermates after 18 weeks of HFD feeding (from 4 to 22 weeks of age). The same cranio-distal depth along the Z-axis of the muscle belly was analyzed in each sample (A,B,E-H). Erk3^f/f^, n = 8 (A-B, E-F), n = 4 (C–D), n = 25 (G–H). Erk3^muscleΔ/Δ^, n = 8 (A–B), n = 4 (C–D), n = 9 (E–F), n = 30 (G–H). Each n represents randomly selected image (A-B,E-H) or different animal (C–D).Data presented as mean ± SEM. ∗p < 0.05, ∗∗p < 0.01, ∗∗∗p < 0.001. Unpaired two-tailed Student's t-test.

To further evaluate the role of Erk3 in fiber-type specification, we performed immunostaining of myosin heavy chain isoforms. HFD typically induces a fiber-type shift in skeletal muscle, characterized by reductions in oxidative type I and IIA fibers and an increase in glycolytic type IIX and IIB fibers, consistent with impaired mitochondrial metabolism and insulin resistance. In line with the protective effect of Erk3 ablation, HFD-fed Erk3^muscleΔ/Δ^ mice exhibited an increased proportion of metabolically intermediate IIA-B hybrid fibers, accompanied by a reduction in glycolytic type IIX fibers (Fig. 6A–B). Consistent with these findings, western blot analysis revealed increased protein levels of myosin heavy chain 2a (MHC-2a), a marker of oxidative fiber types (Fig. 6C–D). Overall, these findings suggest that Erk3 ablation counteracts the HFD-induced fiber-type switch, complementing our results on increased mitochondrial content. In mature, healthy skeletal muscle, nuclei are typically located at the fiber periphery; however, during remodeling or stress, the content of centrally located nuclei increases. Skeletal muscle of HFD-fed Erk3^muscleΔ/Δ^ mice displayed significantly fewer central nuclei compared to controls (Fig. 6E–F), consistent with reduced remodeling and supporting the concept that Erk3 ablation protects against HFD-induced skeletal muscle metabolic impairment. Quantification of myofiber geometry using laminin immunostaining revealed no significant differences in total fiber number, cross-sectional area, Feret diameter, or minimum Feret diameter between HFD-fed Erk3^muscleΔ/Δ^ and control animals (Fig. 6G–H).

## 4 Discussion

Here, we show that targeted deletion of Erk3 in striated muscle is sufficient to protect mice from HFD-induced obesity, glucose intolerance, and insulin resistance, and that such an effect is accompanied by elevated energy expenditure, increased mitochondrial abundance, and a shift toward oxidative muscle fiber identity. This is consistent with our previous findings showing that whole-body loss of Erk3 protects against obesity and improves glucose tolerance and insulin sensitivity [22]. Recently, we also reported that the Erk3-Mk5 complex in adipocytes regulates oxygen consumption and mitochondrial function through FOXO1-dependent mechanisms [13]. Moreover, Soulez et al. showed that inactivation of Erk3 impairs postnatal muscle growth and regeneration through a FOXO3 and MyoD-dependent manner [23]. However, in this study, we did not detect alterations in either total or phosphorylated FOXO1, nor in FOXO3, in skeletal muscle from HFD-fed Erk3^muscleΔ/Δ^ mice. This can be explained by the fact that the mechanism of action of Erk3 depends on the type of tissue and the physiological states, differing under standard chow conditions compared with HFD exposure. We observed the effect of Erk3 ablation in striated muscles on body weight, fat and lean mass, as well as glucose tolerance only when mice were fed HFD. Interestingly, Soulez et al. reported a close to significant decrease in lean mass in mice expressing catalytically inactive Erk3 fed a standard chow diet. One explanation for this discrepancy is the differences in the mouse models. Soulez et al. utilized mice with a global catalytically inactive Erk3 allele, whereas in our study, we used a striated muscle-specific Erk3 knockout.

We showed that striated muscle–specific ablation of Erk3 reduces diet-induced weight gain and adiposity. Our data further suggest that this phenotype may be explained by altered energy expenditure. Although unadjusted energy expenditure exhibited a strong yet non-significant trend toward higher values in HFD-fed Erk3^muscleΔ/Δ^ mice, the effect was most apparent during the dark phase, when mice are typically more active. The difference in nocturnal energy expenditure became significant after normalization to lean mass. Notably, the increase in energy expenditure at rest approached statistical significance. Together with the absence of changes in voluntary activity between groups, this indicates that the effect of Erk3 ablation is independent of locomotor activity. Nevertheless, the impact on energy expenditure remained most evident during the dark phase, suggesting that the metabolic consequences of Erk3 ablation become more pronounced under conditions of increased physiological demand. Collectively, these findings support the interpretation that the phenotype of Erk3^muscleΔ/Δ^ mice reflects enhanced intrinsic metabolic activity of skeletal muscle, particularly unmasked during periods of physical activity. Energy expenditure, food intake, and locomotor activity were measured only in animals fed an HFD. Although Erk3-deficient mice fed a normal diet did not exhibit alterations in body weight or glucose metabolism, even with aging, we cannot exclude the possibility that Erk3 also influences energy expenditure under this dietary regimen. However, we speculate that HFD feeding may exacerbate this difference.

Consistent with this interpretation, we showed that silencing Erk3 or Mk5 in palmitic acid-treated C2C12 myotubes enhances OCR, whereas Erk3 overexpression leads to the opposite effect. Notably, Erk3 knockdown did not alter OCR under basal conditions, while Mk5 silencing increased OCR even in the absence of palmitic acid, suggesting that Erk3 influences mitochondrial respiration primarily under lipid overload, in line with our in *vivo* observation that the Erk3-deficient phenotype becomes apparent only in HFD-fed mice.

Moreover, the Erk3/Mk5-dependent increase in mitochondrial activity was accompanied by greater mitochondrial abundance in C2C12 cells. Similarly, *in vivo* ablation of Erk3 increased OXPHOS complex protein levels, further supporting enhanced mitochondrial function. Interestingly, levels of OXPHOS complexes were significantly increased in the EDL, while only a non-significant trend toward higher OXPHOS content was observed in the quadriceps muscle, and no changes were detected in the soleus. These muscles differ markedly in their fiber-type composition. The EDL consists predominantly of fast-twitch fibers with regional variation between oxidative and glycolytic subtypes [24], whereas the quadriceps muscle group displays a more heterogeneous, mixed fiber-type profile adapted for both endurance and high-force contractions [25]. In contrast, the soleus muscle is enriched in slow-twitch, oxidative type I fibers and displays high baseline mitochondrial content and oxidative capacity [26]. Together, these findings indicate that the effects of Erk3 ablation on mitochondrial content are fiber-type dependent and are preferentially manifested in fast-twitch and mixed skeletal muscles. Of note, mitochondrial abundance and oxidative capacity tightly correlate with fiber-type identity, with oxidative fibers containing substantially more mitochondria than glycolytic fibers [27]. In line with this, transcriptomic and proteomic analyses revealed that silencing Erk3 or Mk5 upregulated genes/proteins characteristic of slow-twitch oxidative fibers, while reducing expression of markers associated with fast-twitch glycolytic fibers. Immunostaining of TA muscle from HFD-fed Erk3^muscleΔ/Δ^ mice confirmed an increase in intermediate type IIA-B hybrid fibers and a trend toward reduced type IIX glycolytic fibers. Consistent with this shift, protein analyses showed a elevation of MHC-2a, isoform expressed in oxidative fibers. Integration of the immunostaining and protein-level results indicates that Erk3 ablation predominantly promotes a shift toward a more oxidative metabolic phenotype in skeletal muscle. A more oxidative muscle phenotype would be expected to be associated with a lower RER. However, no significant differences (only a trend) in RER were observed between HFD-fed Erk3^muscleΔ/Δ^ mice and controls. This likely reflects the fact that both genotypes were maintained on a high-fat diet, which promotes predominant fatty acid utilization and may therefore mask genotype-dependent differences in substrate preference that are more apparent under standard chow conditions.

Importantly, obesity induces a shift in skeletal muscle fiber types characterized by a reduction in oxidative fibers and an increase in more glycolytic fiber types [17]. In the soleus, HFD-induced remodeling involves conversion of type I fibers into faster type I-IIA hybrid fibers, which is related to reduced oxidative capacity and diminished glucose uptake [7]. It has also been reported that prolonged HFD feeding reduces the proportion of type IIA fibers and increases the proportion of type IIX fibers in the transversus abdominis muscle (TA) [28]. These adaptations lead to diminished muscle oxidative metabolism, impaired lipid utilization, and reduced glucose uptake. Importantly, glycolytic fibers express lower levels of the glucose transporter GLUT4 and exhibit decreased insulin responsiveness compared to oxidative fibers, thereby diminishing the muscle's capacity to clear glucose from the circulation and promoting hyperglycemia [29]. Moreover, a reduced oxidative capacity restricts fatty acid oxidation, facilitating intracellular lipid accumulation and further exacerbating insulin resistance and metabolic dysfunction [30]. In line with this, silencing Erk3 or Mk5 in myotubes reduced the expression of the lipid droplet-associated protein PLIN2, whose abundance negatively correlates with skeletal muscle fatty acid-oxidative capacity and hypertrophic growth in mice [31], whereas elevated PLIN2 levels have been linked to muscle atrophy [32]. Furthermore, Erk3 ablation decreases the proportion of centrally located nuclei, which in HFD-fed mice typically reflect chronic muscle stress and ongoing remodeling [33]. This, together with the fact that Erk3 ablation promotes a shift toward a more oxidative metabolic phenotype in skeletal muscle, suggesting that Erk3 ablation protects from HFD-induced skeletal muscle remodeling and fiber switch, and as a consequence, an impaired glucose homeostasis. This is in line with our results showing that HFD-fed Erk3^muscleΔ/Δ^ mice are more glucose and insulin tolerant.

Tesnière et al. recently showed that intracellular acidification markedly increases Erk3 stability, whereas alkalinization accelerates its degradation [34]. This is consistent with the fact that glycolytic muscle fibers generate substantially more lactate, and therefore stronger intracellular acidification, suggesting that the shift toward more oxidative fibers upon Erk3 loss aligns with reduced reliance on glycolytic, lactate-producing metabolism. Interestingly, the intracellular pH does not affect in the same way Mk5 [34], underscoring intrinsic differences in their regulatory dynamics. In this regard, it is worth noting that, although as we demonstrated, Mk5 contributes to Erk3 stabilization in skeletal muscle cells, our transcriptomic and proteomic analyses show that the two kinases control largely distinct sets of targets with relatively little overlap. This divergence supports the conclusion that, despite the broadly similar phenotypes observed upon silencing either kinase, the underlying molecular programs they control are not identical. All these together indicate that Erk3 and Mk5 may also associate with other protein partners that differentially shape their stability and function.

The transcriptomic and proteomic signatures identified here show that Erk3 depletion in C2C12 cells influences not only muscle-specific pathways but also developmental, signaling, and immune or stress-responsive programs, whereas Mk5 loss is associated with enrichment of transport- and stress-related processes. These patterns, however, likely reflect the limitations of the C2C12 myotube system, which represents a reductionist *in vitro* model and does not fully mirror the genetic and physiological complexity of skeletal muscle *in vivo,* as they differentiated mostly into fast-twitch type II fibers [35,36].

It is important to note that Erk3 deletion in this model targets striated muscle and therefore also affects cardiac tissue. Consistent with this, we observed a reduction in Erk3 protein levels in the heart; however, this decrease was less pronounced than in skeletal muscle, likely reflecting the higher cellular heterogeneity of cardiac tissue.

In conclusion, our study identifies Erk3 as a regulator of skeletal muscle oxidative phenotype and whole-body metabolic homeostasis. Muscle-specific Erk3 deletion enhances mitochondrial function, shifts fiber identity toward oxidative types, and protects against HFD-induced obesity and insulin resistance. These findings highlight Erk3 as a promising therapeutic target for improving skeletal muscle metabolism and treating obesity-associated metabolic disorders.

## CRediT authorship contribution statement

**Angel Loza-Valdes:** Writing – review & editing, Visualization, Validation, Project administration, Methodology, Investigation, Formal analysis, Conceptualization. **Carlos Acosta-Gallo:** Writing – review & editing, Visualization, Methodology, Investigation, Formal analysis, Conceptualization. **Toufic Kassouf:** Visualization, Methodology, Investigation, Formal analysis. **Andrei Belykh:** Methodology, Investigation. **Małgorzata Stelmach:** Investigation. **Dominika Malińska:** Writing – review & editing, Investigation. **Katia El Ghoz:** Investigation. **Rabih El-Merahbi:** Investigation. **Filip Dziaczkowski:** Investigation. **Katarzyna Kolczyńska-Matysiak:** Writing – original draft, Visualization, Validation, Supervision, Methodology, Formal analysis, Conceptualization. **Grzegorz Sumara:** Writing – review & editing, Supervision, Resources, Project administration, Investigation, Funding acquisition, Conceptualization.

## Funding

This work was supported by the National Science Centre, Poland (Sonata Bis 10, grant no. UMO-2020/38/E/NZ4/00314). GS, KKM, and TK are additionally supported by the Dioscuri Centre of Scientific Excellence Grant (UMO-2018/01/H/NZ4/00002) - the program initiated by the Max Planck Society (Max-Planc-Gesellschaft), managed jointly with the National Science Centre in Poland (Narodowe Centrum Nauki) and mutually funded by the Polish Ministry of Science and Higher Education (Ministerstwo Nauki i Szkolnictwa Wyższego) and the German Federal Ministry of Education and Research (Bundesministerium für Bildung und Forschung). ALV was supported by an EMBO installation grant (No 4425) awarded to GS.

## Declaration of competing interest

The authors declare that they have no known competing financial interests or personal relationships that could have appeared to influence the work reported in this paper.

## Data Availability

The RNA-seq data generated in this study have been deposited in the Gene Expression Omnibus (GEO) under accession number GSE319950. The mass spectrometry proteomics data have been deposited to the ProteomeXchange Consortium via the PRIDE partner repository with the dataset identifier PXD074086. All remaining source data reported in this paper have been archived in the Open Science Framework (OSF) repository under DOI: 10.17605/OSF.IO/T382E and are publicly available.
