# Supplementary material for: Erk3 deletion drives oxidative adaptations in skeletal muscle
Source: Mol Metab. 2026 Feb 24;106:102338. doi: 10.1016/j.molmet.2026.102338 (PMC12969653; doi:10.1016/j.molmet.2026.102338)
Supplement: Multimedia component 1 [file mmc1.docx]

**Supplementary Figure 1**


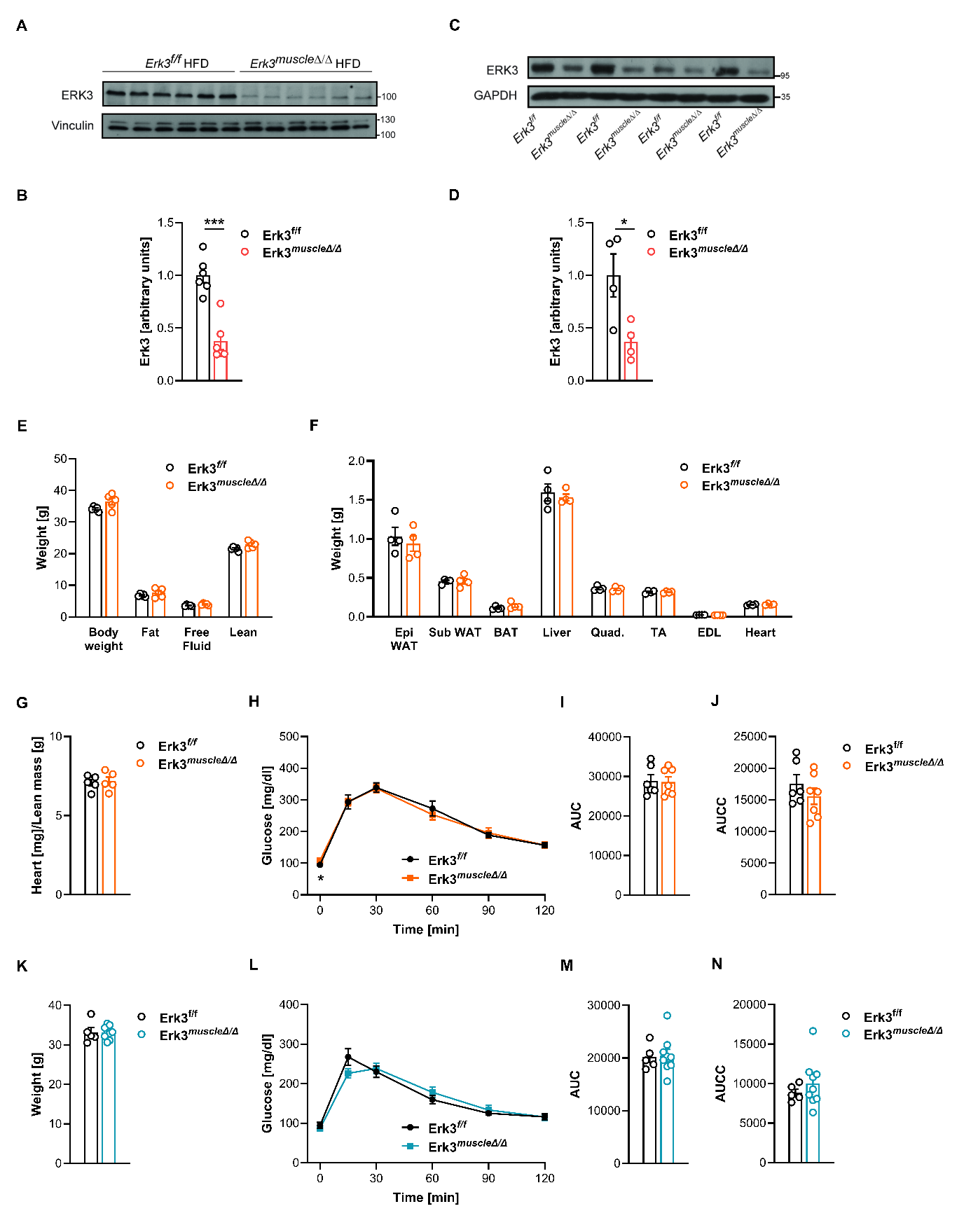


**Supplementary Figure 2**


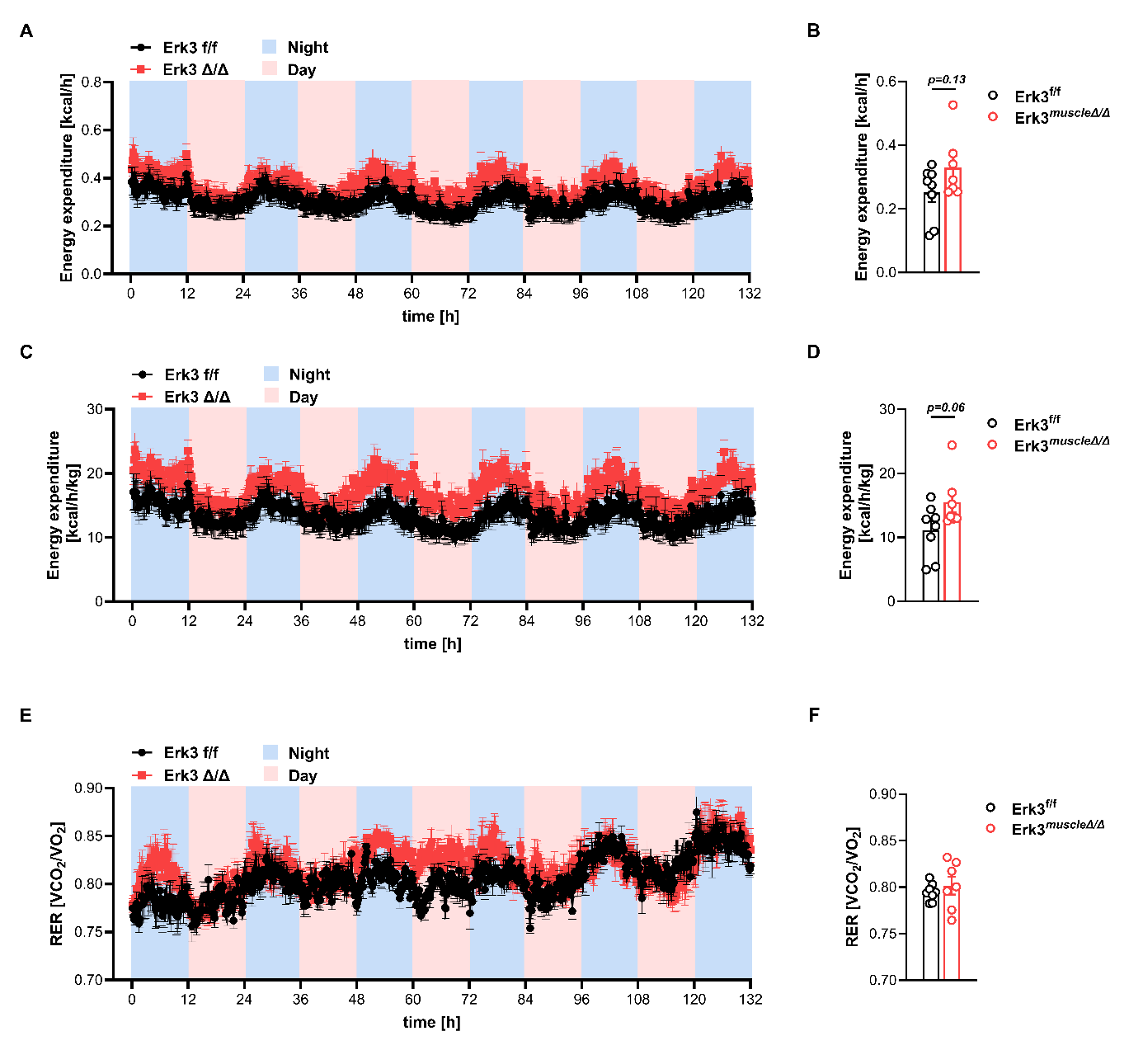


**Supplementary Figure 3**


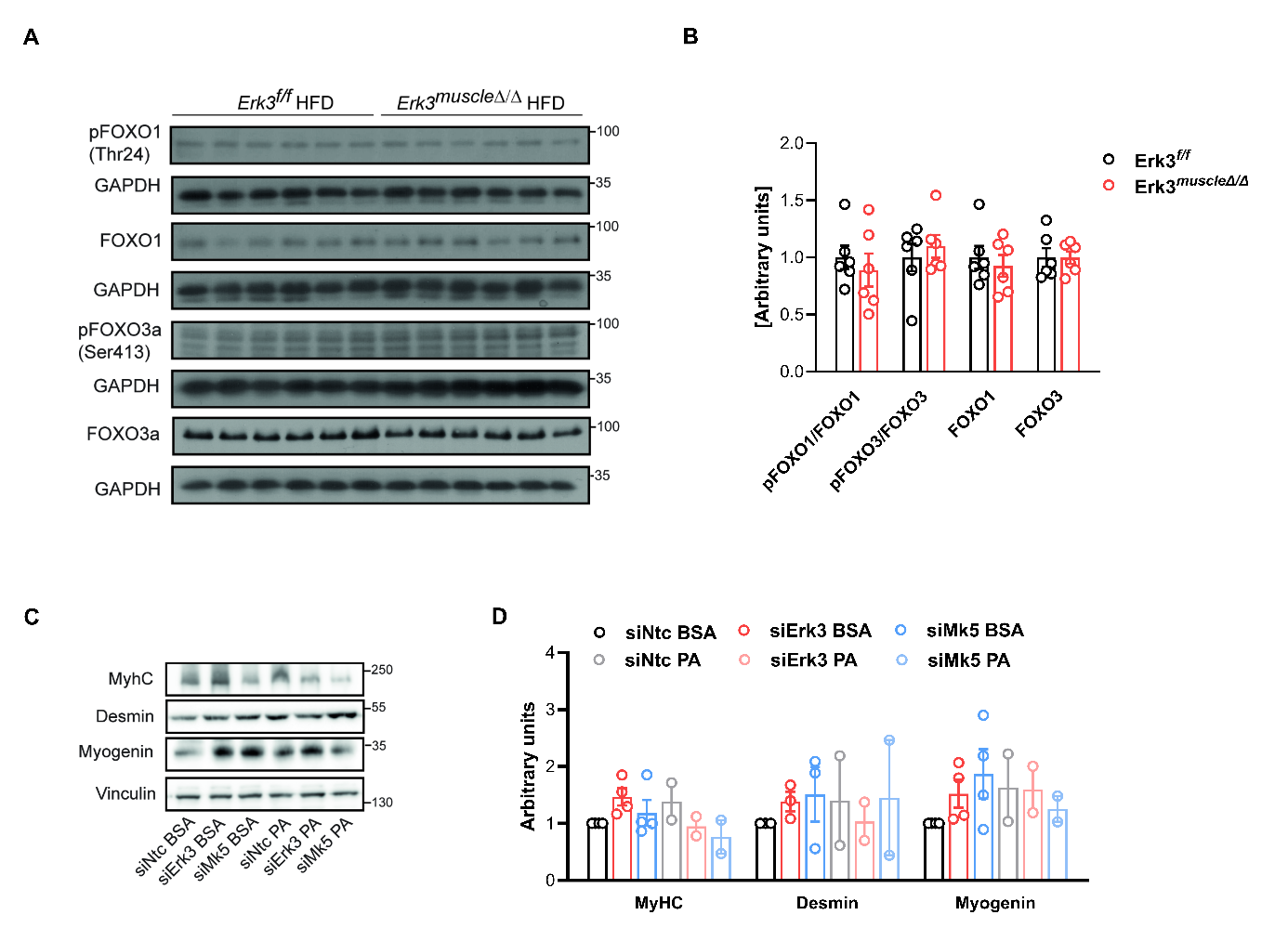


**Supplementary Figure 4**


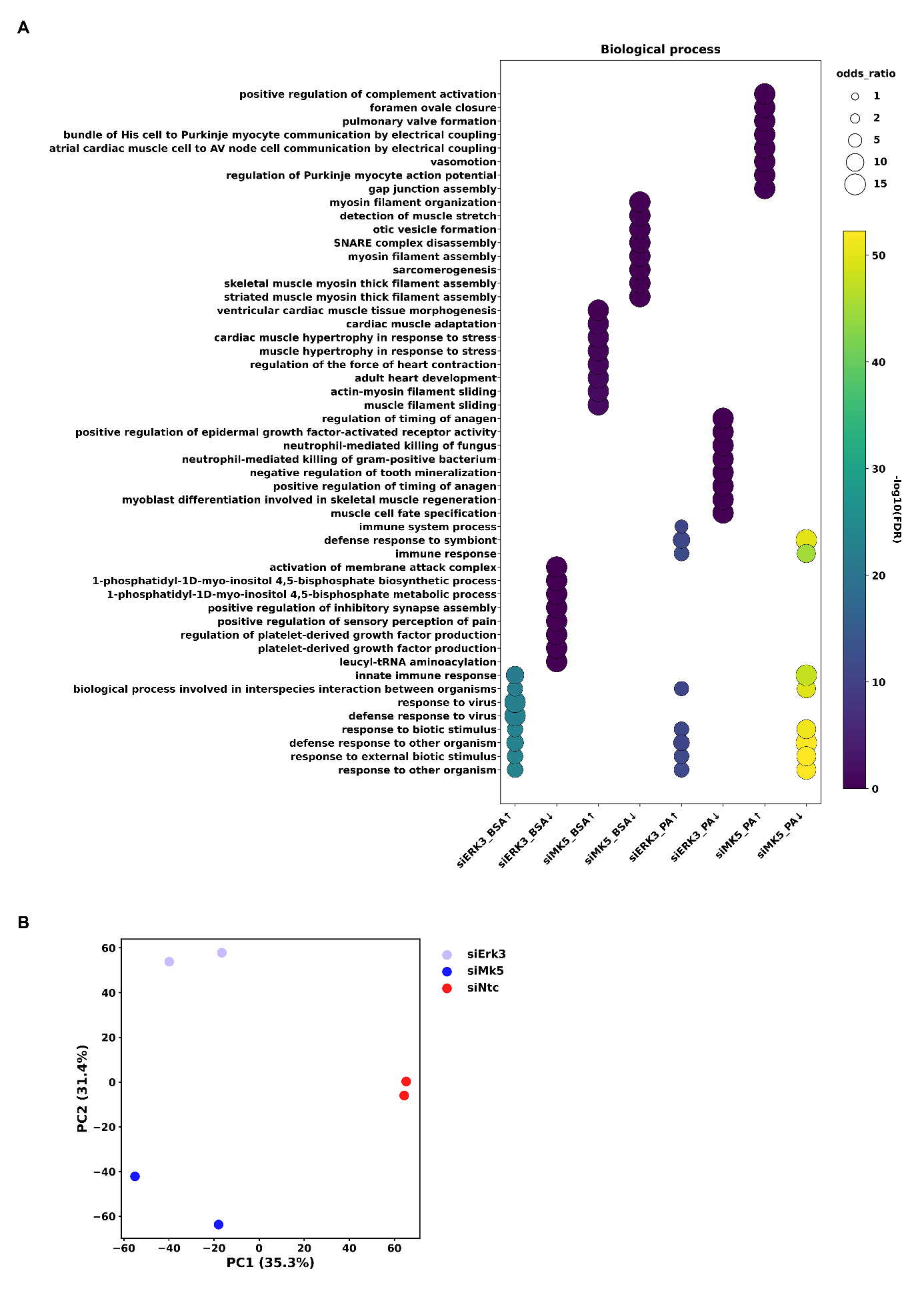


**Figure S1. Striated muscle-specific Erk3 ablation does not affect body mass and glucose tolerance test in mice fed a standard chow diet.**

Representative immunoblots (A,C) and quantification (B,D) of Erk3 in tibialis anterior muscle (A-B) and heart (C-D) from Erk3^muscleΔ/Δ^ mice and control littermates Erk3^f/f^ after 18 weeks of HFD feeding (from 4 to 22 weeks of age). Body composition (E), organ and tissue weight (F,G), and glucose tolerance test (H-J) in Erk3^muscleΔ/Δ^ mice and control littermatesErk3^f/f^ fed a standard chow diet at 22 weeks of age. Body weight (K) and glucose tolerance test (L-N) in Erk3^muscleΔ/Δ^ mice and control littermates Erk3^f/f^ fed a standard chow diet at 71 weeks of age. Erk3^muscleΔ/Δ^, n=6 (A-B), n=4 (C-D,F), n=5 (E,G), n=7 (H-J), n=9 (K-N). Erk3^f/f^, n=6 (A-B, H-J), n=4 (C-D,F), n=5 (E,G,K-N). AUC - total area under the curve, AUCC - incremental area under the curve. Each n represents a sample from distinct mice. Data presented as mean ±SEM.

**Figure S2. Effect of Erk3 ablation on metabolism in skeletal muscle.**

Unnormalized (A-B) and lean mass-normalized (C-D) energy expenditure, as well as respiratory exchange ratio (E-F) monitored over a prolonged period across the diurnal cycle (A,C,E) or at rest (B,D,F) in Erk3^muscleΔ/Δ^ mice (n=7) and control littermates Erk3^f/f^ (n=8) after 18 weeks of HFD feeding (from 4 to 22 weeks of age). RER - respiratory exchange ratio. Each n represents a sample from distinct mice. Data presented as mean ±SEM. *p<0.05. Unpaired two-tailed Student’s t-test.

**Figure S3. Effect of Erk3 ablation on FOXO proteins and skeletal muscle differentiation markers.**

Representative immunoblots (A,C) and quantification (B,D) of indicated proteins in tibialis anterior muscle of Erk3^muscleΔ/Δ^ (n=6) and control littermates Erk3^f/f^ (n=6) after 18 weeks of HFD feeding (from 4 to 22 weeks of age) (A-B), and C2C12 cells after Erk3 (siErk3) or Mk5 (siMk5) silencing under BSA or 0.5 mM palmitic acid (PA) treatment for 16 h (C-D). siNtc - cells treated with non-targeting siRNA. Each n represents a sample from distinct mice (A-B) or independent biological replicate (C-D). Data presented as mean ±SEM. *p<0.05. Unpaired two-tailed Student’s t-test.

**Figure S4. Erk3 and Mk5 silencing alter various biological processes in C2C12 myotubes.**

Functional enrichment analysis using g:Profiler (GO:BP) of significantly regulated genes identified in transcriptomic datasets (A), and PCA analysis of significantly regulated proteins identified in proteomic datasets (B) from C2C12 cells following Erk3 (siErk3) or Mk5 (siMk5) silencing after 16h treatment with BSA (A-B) or 0.5 mM palmitic acid (PA) (A).
